# Circulatory and Urinary B-Vitamin Responses to Multivitamin Supplement Ingestion Differ between Older and Younger Adults

**DOI:** 10.3390/nu12113529

**Published:** 2020-11-17

**Authors:** Pankaja Sharma, Soo Min Han, Nicola Gillies, Eric B. Thorstensen, Michael Goy, Matthew P. G. Barnett, Nicole C. Roy, David Cameron-Smith, Amber M. Milan

**Affiliations:** 1 The Liggins Institute, University of Auckland, Auckland 1023, New Zealand; p.sharma@auckland.ac.nz (P.S.); clara.han@auckland.ac.nz (S.M.H.); n.gillies@auckland.ac.nz (N.G.); e.thorstensen@auckland.ac.nz (E.B.T.); michael_goy@waters.com (M.G.); dcameron_smith@sics.a-star.edu.sg (D.C.-S.); 2 Riddet Institute, Palmerston North 4474, New Zealand; matthew.barnett@agresearch.co.nz (M.P.G.B.); nicole.roy@otago.ac.nz (N.C.R.); 3 Food & Bio-based Products Group, AgResearch, Palmerston North 4442, New Zealand; 4 High-Value Nutrition National Science Challenge, Auckland 1023, New Zealand; 5 Department of Human Nutrition, University of Otago, Dunedin 9016, New Zealand; 6 Singapore Institute for Clinical Sciences, Agency for Science, Technology, and Research, Singapore 117609, Singapore

**Keywords:** B-vitamins supplement, vitamin B_6_, ageing, B-vitamin bioavailability, ultra-high performance liquid chromatography coupled with mass spectrometry, excretion

## Abstract

Multivitamin and mineral (MVM) supplements are frequently used amongst older populations to improve adequacy of micronutrients, including B-vitamins, but evidence for improved health outcomes are limited and deficiencies remain prevalent. Although this may indicate poor efficacy of supplements, this could also suggest the possibility for altered B-vitamin bioavailability and metabolism in older people. This open-label, single-arm acute parallel study, conducted at the Liggins Institute Clinical Research Unit in Auckland, compared circulatory and urinary B-vitamer responses to MVM supplementation in older (70.1 ± 2.7 y, *n* = 10 male, *n* = 10 female) compared to younger (24.2 ± 2.8 y, *n* = 10 male, *n* = 10 female) participants for 4 h after the ingestion of a single dose of a commercial MVM supplement and standardized breakfast. Older adults had a lower area under the curve (AUC) of postprandial plasma pyridoxine (*p* = 0.02) and pyridoxal-5′phosphate (*p* = 0.03) forms of vitamin B_6_ but greater 4-pyridoxic acid AUC (*p* = 0.009). Urinary pyridoxine and pyridoxal excretion were higher in younger females than in older females (time × age × sex interaction, *p* < 0.05). Older adults had a greater AUC increase in plasma thiamine (*p* = 0.01), riboflavin (*p* = 0.009), and pantothenic acid (*p* = 0.027). In older adults, there was decreased plasma responsiveness of the ingested (pyridoxine) and active (pyridoxal-5′phosphate) forms of vitamin B_6_, which indicated a previously undescribed alteration in either absorption or subsequent metabolic interconversion. While these findings cannot determine whether acute B_6_ responsiveness is adequate, this difference may have potential implications for B_6_ function in older adults. Although this may imply higher B vitamin substrate requirements for older people, further work is required to understand the implications of postprandial differences in availability.

## 1. Introduction

B-vitamins are indispensable for human health given their roles in cell metabolism. These water-soluble vitamins exist in various coenzyme forms (vitamers) which are essential for normal function of enzymes linked to energy metabolism in the tricarboxylic acid (TCA) cycle [1], and in the one carbon metabolism pathway for DNA synthesis and repair, epigenetic regulation, and homocysteine regulation [2]. While the central roles of folate and vitamin B_12_ in regulating one-carbon metabolism are well described [3], the B_6_-vitamer pyridoxal 5’-phosphate (PLP) is also required for the irreversible transsulfuration of homocysteine to cysteine, a precursor of the antioxidant glutathione [4], as well as being important in cellular protein metabolism [5]. Thiamine, riboflavin, niacin, pantothenic acid, and biotin are precursors of coenzymes in mitochondrial energy synthesis [1]. Hence, it is well recognized that an adequate dietary intake of B-vitamers is fundamentally important for cellular functioning and health maintenance, including cognitive function [6], mediation of systemic acute phase inflammation [7,8], and cardiovascular health [9].

Nutritional sufficiency is an increasing challenge for the growing global aging population [10,11]. The aging process can either adversely impact nutrient intake and/or impair digestive function, which collectively increases the risks for micronutrient inadequacy [12]. In addition, some water-soluble B-group vitamins inherently have limited absorption [13], are transient in the body [14,15], and are excreted in the urine [16]. Decreased dietary intake in aging is therefore widely assumed to be the major the risk of deficiency [17,18]. However, there is also evidence that increased malabsorption or impaired metabolism, well documented for vitamin B_6_ and B_12_, may elevate the risks for deficiency [13,19,20].

Although consuming a healthy varied and balanced diet is encouraged by organizations including the U.S. Department of Agriculture (USDA) [21] and the New Zealand Ministry of Health [22], many seniors regularly consume multivitamin and mineral supplements (MVM) [23] or choose vitamin fortified foods with the aim of improving health status [24]. Yet, the efficacy of MVMs in older populations remains controversial [10]. Higher doses of B-vitamins may be necessary for older adults to normalize status [25,26], which may similarly point towards an altered bioavailability of the ingested B-vitamins with advancing age. However, it remains unclear whether the postprandial availability of B-vitamins is altered with increasing age. Post-ingestion vitamin B_6_ bioavailability has previously been shown not to differ with age [13]. Most acute investigations have been undertaken in younger individuals [27,28], and provide limited information regarding acute postprandial responses of the range of B-vitamin and vitamers in healthy older people. In addition to implications for MVM efficacy, any age-related postprandial alterations may have relevant implications to postprandial metabolic flux such as glucose homeostasis, one carbon metabolism [29], or cognitive function [14].

The present study examined the postprandial responses of a wide range of B vitamins and vitamers to a single MVM supplement ingested with a test meal in older compared to younger adults. We hypothesized that postprandial vitamin B_12_ and B_6_ availability is less responsive in healthy older adults, Plasma and urine samples were assessed, and men and women included to identify any sex-specific effects. A profile of 14 vitamins and vitamers was achieved using a recently validated ultra-high performance liquid chromatography coupled with mass spectrometry (UHPLC-MS/MS) technique [30] complemented by immunoenzymatic methods.

## 2. Materials and Methods

### 2.1. Study Design

The study was an open-label, single-arm acute parallel trial. Participants presented to the clinic after an overnight fast to ingest a single tablet of MVM (Centrum Advance General Multi, Pfizer, New York, NY, USA) along with a standardized breakfast. Blood samples were collected at fasting and hourly for 4 h following the meal and supplement. Urine was collected at fasting and all subsequent urine produced during the 4 h.

### 2.2. Participants

The participants, 20 younger adults and 20 older adults, were recruited from the community through advertisements internal to the University of Auckland, in local newspapers in Auckland, New Zealand, and via social media. An equal number of males and females were recruited. Eligible participants were required to be between age ranges 19–30 years or 65–76 years, healthy, with a BMI ranging from 18–30 kg/m^2^, with no major medical conditions and non-smokers. Exclusion criteria included: current consumption of multivitamin and mineral supplements (within 3 weeks); any present or recent history of gastrointestinal diseases including celiac, Crohn’s, colitis; medical history of myocardial infarction, angina, stroke, or cancer; pre-existing metabolic disease or diabetes; self-reported alcohol intake > 28 units per week. Similarly, individuals with food allergies or intolerances to the intervention foods or who were taking any medications likely to impact on digestive or metabolic function, including proton pump inhibitors and thyroid medications, were excluded.

### 2.3. Ethics Approval

Ethics approval was obtained from The University of Auckland Human Participants Ethics Committee (UAHPEC; Reference No. 019392). The trial was registered prospectively at Australia New Zealand Clinical Trials Registry (ANZCTR) ID: ACTRN12617000969369. Written informed consent was obtained from all participants. The clinical trial was conducted between July-September 2017 at the Clinical Research Unit (CRU) based at the Liggins Institute, the University of Auckland.

### 2.4. Intervention: Standardized Breakfast Meal

The breakfast items were purchased from a local supermarket (Countdown, Progressive Enterprises, Auckland, New Zealand) and prepared onsite prior to the trial day. The breakfast meal consisted of two slices of white bread (74 g prior to light toasting), butter (10 g), honey (20 g), apple sauce (100 g), and orange juice (250 mL), based on a previously described meal [31]. Participants were instructed to consume all of the items provided. Unconsumed items were weighed and recorded. The amounts of each food item consumed by the participants are presented in Table 1 and the estimated intakes of B-vitamins from the supplement and the test meal are presented in Table 2. On average, the younger adults left more unconsumed portions of honey and applesauce compared to the older adults (*p* = 0.02, for both).

### 2.5. Outcomes

The study was powered to investigate the maximum concentration (C_max_) of B_12_ between younger and older subjects, while also investigating the postprandial responses of vitamin B_1_ (thiamine), B_2_ (riboflavin), B_3_ (niacin), B_5_ (pantothenic acid), B_6_ (pyridoxine), B_7_ (biotin), folic acid and their derivatives in plasma, serum, and urine. Other secondary outcomes, not reported here, were postprandial circulating levels of plasma minerals, metabolites, and urinary minerals and metabolites.

#### 2.5.1. Anthropometric Measurements

Anthropometric measurements, including body weight, height, waist circumference, and resting blood pressure, were recorded as an average of two measurements. Body weight was measured to the nearest 0.1 kg with light clothing and without shoes using a digital weighing scale (Wedderburn WM206, Taiwan). Height was measured to the nearest 0.1 cm without shoes using a stadiometer (Holtain Ltd., Crymych, Dyfed, UK), waist circumference was measured using a non-extensible measuring tape following an international standardized protocol [32] and resting blood pressure was measured using an automatic blood pressure monitor (Heart Sure BP100, Omron, Kyoto, Japan).

**Table 2:** Estimated B-vitamin intake from a single multivitamin tablet and the standard breakfast test meal by the study participants.

Vitamins	Supplement	Test Meal	Total Intake
Vitamin B_12_ (µg)	22.00	0.00	22.00
Total Folate (µg)	0.00	79.01	79.01
Folic Acid (µg)	400.00	0.00 ^1^	400.00
Vitamin B_6_ (mg)	6.00	0.38	6.38
Thiamine (mg)	2.18	0.43	2.61
Pantothenic acid (mg)	10.80	0.00 ^1^	10.80
Riboflavin (mg)	3.20	0.16	3.36
Niacin (mg)	15.00	0.78	15.78
Biotin (µg)	45.00	0.00 ^1^	45.00

B-vitamin content of multivitamin supplement tablet was obtained from nutrition information available from Centrum (Pfizer, New York, NY, USA). Foodworks software was used to extract the nutrient composition of test meals from the New Zealand food composition database. ^1^ Values not present in the database [33]; folic acid was not present as fortified foods were not present in the test meals.

#### 2.5.2. Dietary and Meal Intake Analysis

Dietary B-vitamin intake was determined from self-reported 3-day food dairy records. Food records and test meals were analyzed by a New Zealand Registered Dietitian using Foodworks 8 Professional (Xyris Software PTY Ltd., Brisbane, Australia) using the New Zealand Food Composition Database (FOODfiles™ 2016 Version 1).

#### 2.5.3. Biochemical Measures

Serum glucose and triglyceride, fasting cholesterol, low density lipoprotein cholesterol (LDL-C), high density lipoprotein cholesterol (HDL-C), and urinary creatinine measurements were performed using a Roche Cobas c311 clinical chemistry autoanalyzer (Roche Diagnostics GmbH, D-68298 Mannheim, Germany) based on enzymatic colorimetric assays. Serum insulin was measured using a Roche Cobas e411 autoanalyzer (Roche Diagnostics, Mannheim, Germany) utilizing an electrochemiluminescence immunoassay.

#### 2.5.4. Analysis of B-Vitamins

All blood samples were collected in light-protected ethylenediaminetetraacetic acid (EDTA) vacutainers for plasma and additive-free vacutainers for serum. Serum tubes were left to clot at room temperature for 15 min and both plasma and serum tubes were centrifuged at 1500× *g* for 15 min at 4 °C. Aliquots were stored in amber vials at −80 °C until analysis.

Vitamin B_12_ and folate were measured in serum samples using the Roche Cobas e411 autoanalyzer. These assay methods were not optimized for analysis in urine samples, nor are these vitamin forms standardly measured in urine [34], so they were only applied to serum samples.

All other B-vitamins and vitamers were measured using UHPLC-MS/MS with slight modifications to the previously published method [30]. This included thiamine, riboflavin and its vitamer flavin mononucleotide (FMN), nicotinic acid (niacin) and its vitamers (nicotinamide and nicotinuric acid), pantothenic acid, vitamin B_6_ vitamers (pyridoxine, pyridoxal, pyridoxamine, PLP, and 4-pyridoxic acid (4-PA) in plasma), and urine. Biotin and folic acid concentrations were also measured in urine samples. The technical details of plasma samples preparation and UHPLC-MS/MS analysis have been reported elsewhere [35]. In brief, an automated liquid handling robotic system Eppendorf epMotion^®^ 5075 automated liquid handling system (Eppendorf, AG, Hamburg, Germany) was used for plasma sample preparation. Plasma protein was precipitated using 400 μL of methanol containing 0.3% acetic acid and 2.5% H_2_O, pipetted into a 2 mL square 96-well Impact^TM^ Protein Precipitation filter plate (Phenomenex, Torrance, CA, USA). As certified reference materials were not available for this assay, matrix-matched quality controls of pooled human plasma spiked with known concentrations of the vitamins and vitamers of interest were used as quality controls (QCs) [30]. All samples, QCs, and standards were spiked with 10 μL of internal standard mix, mixed thoroughly, and filtered into a 96-well square collection plate by applying vacuum pressure using the robot. This was followed by solvent evaporation and reconstitution of the concentrated samples with 200 µl reconstitution solvent made up of water containing 5% acetic acid, 0.2% heptafluorobutyric acid, and 1% ascorbic acid, then placed into the autosampler of the UHPLC-MS/MS for injection. Urine samples were diluted 10-fold with the reconstitution solvent prior to centrifugation and injection to capture the measures within the calibration range. The urinary concentrations of B-vitamins were reported per urinary creatinine concentration to account for differences in urine output.

### 2.6. Sample Size, Data Interpretation, and Statistical Methods

There are limited data reporting acute vitamin bioavailability across a range of B-vitamins and vitamers, as previous studies have primarily focused on vitamin B_12_, vitamin B_6_, and folate [14], yet expected ranges for vitamers are scarce. Further, few comparisons between younger and older subjects exist. Sample size calculations were therefore based on published data of supplemental B_12_ bioavailability; an expected C_max_ after ingestion is 549 ± 128 pg/mL [27]. A sample size of 20 subjects per group was estimated for a between group difference of 20% to identify significant differences (α = 0.05 and β = 0.8) in the proposed primary outcome measure (vitamin B_12_).

Data are presented as means ± SEM. The incremental area under the curve (AUC) was obtained after correction for baseline concentrations. Homeostatic model assessment of insulin resistance (HOMA-IR) was calculated from fasting glucose and insulin concentrations using the equation from Matthews et al. [36] Statistical significance was tested using SPSS (IBM SPSS Statistics 25). To determine the effects of time, a general linear mixed model was used with time as repeated measures and group (age) and sex as fixed factors. All other age and sex comparisons were done using the general linear model. In case of an interaction, post hoc tests were performed using Sidak–Holm adjustments for pairwise comparisons. The alpha was set at 0.05.

## 3. Results

### 3.1. Baseline Characteristics

**Figure 1:**
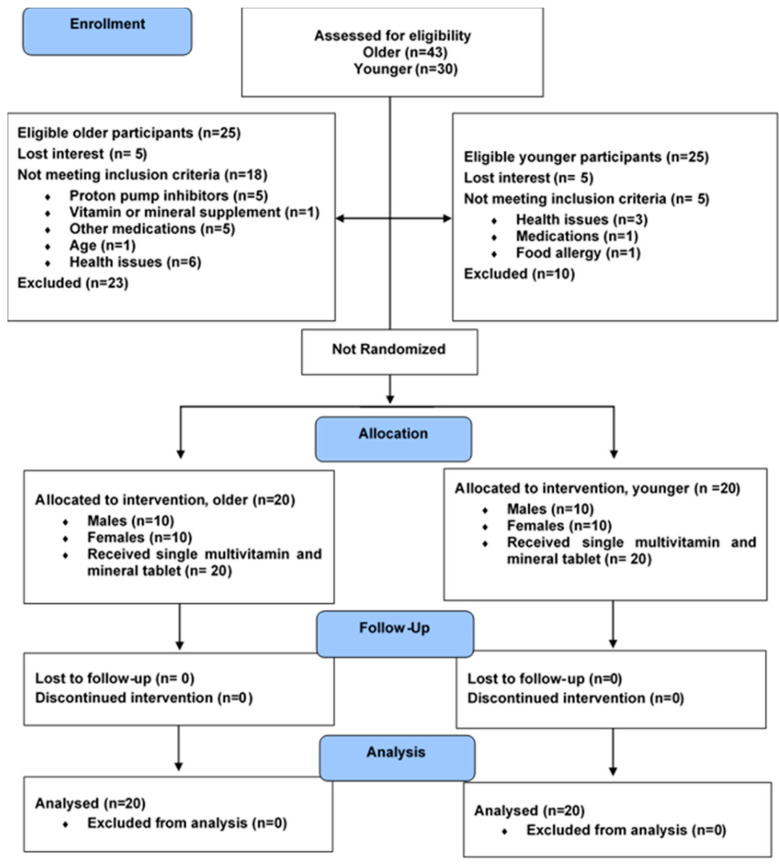
Consort flow diagram of study participant recruitment, intervention, follow-up, and analysis.

A total of 40 participants completed the study; *n* = 20 older, *n* = 20 younger (Figure 1 and Table 3). The average age (mean ± SEM) of younger and older adults was 24.2 ± 0.6 and 70.1 ± 2.5 years, respectively. Older subjects had higher systolic and diastolic blood pressure, fasting glucose, LDL-C, total cholesterol, and triglycerides (*p* < 0.01), compared to the younger group. However, HDL-C, insulin, and HOMA-IR did not differ by age.

### 3.2. Estimated B-Vitamin Intake from 3-Day Food Record

Generally, the mean vitamin intake of participants met the recommended dietary intake (RDI); the exceptions were dietary folate equivalents (DFEs) and thiamin in younger females. Older individuals had lower habitual vitamin B_12_ intake than younger adults (main age effect, *p* = 0.003; Table 4). Differences in intake for many B-vitamins between the older and younger adults were sex-dependent. Total folate, vitamin B_6_, riboflavin, and niacin intakes were higher in younger males (age × sex interaction, *p* < 0.05) compared to all other groups. Regardless of age, males had greater thiamine intake than females (main sex effect, *p* = 0.009).

### 3.3. Urine Output and Creatinine Concentration

Urine volume, both at fasting and postprandially (accumulated over 1–4 h) during the trial period, was not different between any age or sex groups (*p* > 0.05, Supplemental Table S1). However, fasted urinary creatinine concentrations were greater in younger than older adults (main age effect, *p* = 0.005), and the postprandial concentration was greater in males compared to females (main sex effect, *p* = 0.012).

### 3.4. Fasting Circulating Vitamin Status and Urinary Concentrations

Fasting serum B_12_ concentrations were different between younger and older adults, but this difference was sex-specific (Table S2), with lower concentrations in older males (age × sex interaction, *p* = 0.005) than younger males (*p* = 0.002) or older females (*p* = 0.009). Older compared to younger individuals had higher serum folate (16.2 ± 1.7 vs. 9.5 ± 0.8 mg/mL) and plasma thiamine (1.7 ± 0.3 vs. 0.6 ± 0.1 nmol/L) concentrations at baseline (main age effect, *p* = 0.001 and *p* < 0.001, respectively). Fasting plasma pantothenic acid concentration was higher in older females (age × sex interaction, *p* = 0.014) than younger females (*p* = 0.004) and older males (*p* = 0.009). Fasting plasma concentration of nicotinuric acid, the excretory metabolite of vitamin B_3_, was lower in older females (age × sex interaction, *p* = 0.016) compared to younger females (*p* = 0.014) but was not different significantly between old and young males or between sexes. Fasting urinary riboflavin concentration was higher in older compared to younger adults (337.8 ± 41.5 vs. 188.7 ± 39.8 nmol/mmol creatinine; main age effect, *p* = 0.015) (Table S2), whereas the riboflavin-vitamer FMN was not different. Similarly, older adults had greater urinary nicotinic acid compared to younger adults (82.7 ± 10.1 vs. 47.1 ± 6.1 nmol/mmol creatinine; main age effect, *p* = 0.003), whereas nicotinamide and nicotinuric acid concentrations differed between age groups depending on participant sex (age × sex interactions *p* < 0.05, Table S2). Younger females excreted more nicotinuric acid than older females (*p* = 0.007) and more nicotinuric acid and nicotinamide than younger males (*p* = 0.004 and *p* = 0.013, respectively). No other measured vitamins or vitamers differed between older and younger adults, but several sex differences were observed at fasting (Table S2).

### 3.5. Acute Postprandial Response of B-Vitamins and Vitamers Following MVM Supplement Ingestion

**Figure 2:**
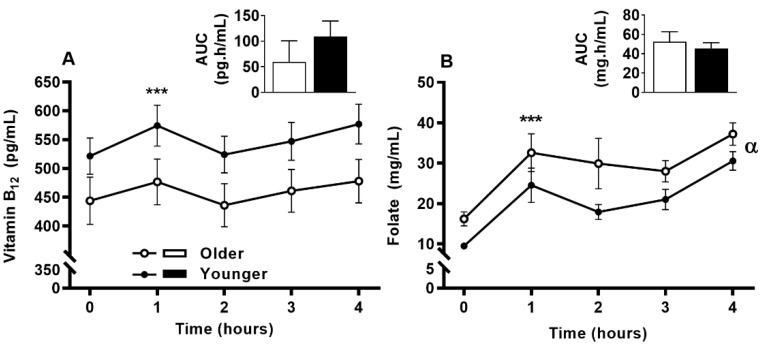
Serum (**A**) vitamin B_12_ and (**B**) folate at fasting (time, 0), hourly until 4 h (time, 1, 2, 3, 4) and the four hour incremental area under the curve (AUC, represented by bar-graphs) following a single multivitamin and mineral supplement ingestion in older and younger adults. Data for multiple time points was compared using general linear mixed model with time as repeated and age as fixed factor. AUC data was compared with general linear univariate analysis of variance with age as fixed factor. There was a main time effect for serum vitamin B_12_ concentration (*p* < 0.001) and main time (*p* < 0.001) and main age effect (*p* = 0.022) for serum folate. Both vitamin B_12_ and folate AUC were not different between age groups. ***, *p* < 0.001, significant increase following supplement intake relative to baseline in both age groups; α, *p* < 0.05, main age effect.

**Figure 3:**
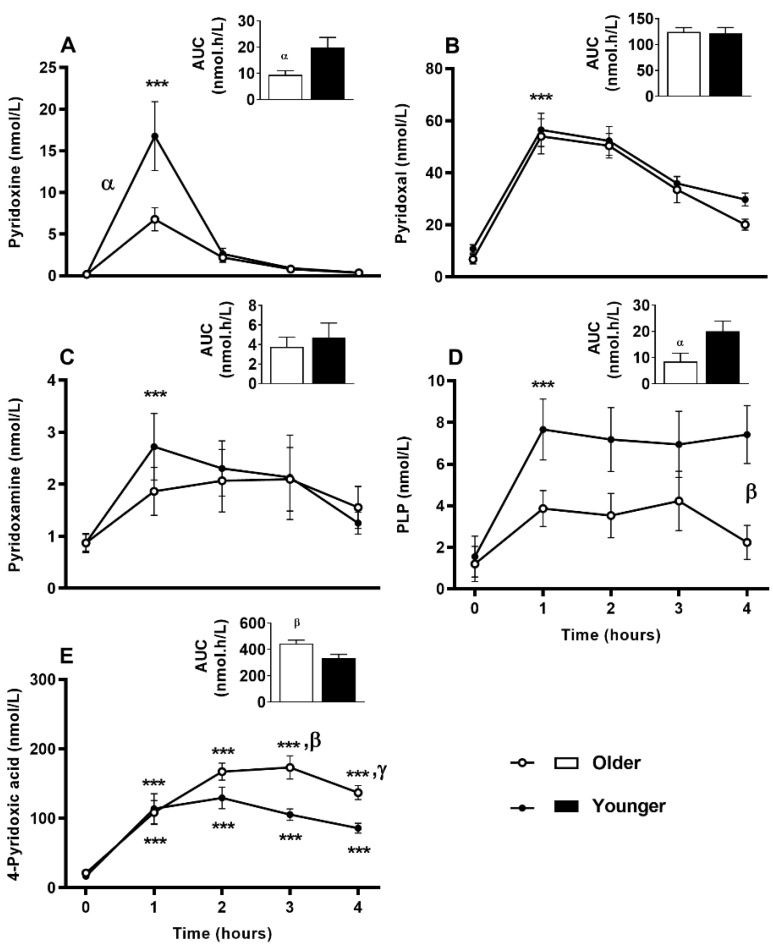
Plasma response of B_6_ vitamers at fasting (time, 0), hourly until 4 h (time, 1, 2, 3, 4) and the four hour incremental area under the curve (AUC, represented by bar-graphs) following a single multivitamin and mineral supplement ingestion in older and younger adults. (**A**), Pyridoxine (**B**), pyridoxal (**C**), pyridoxamine (**D**), pyridoxal 5′-phosphate (PLP), and (**E**) 4-pyridoxic acid. Data for multiple time points was compared using general linear mixed model with time as repeated and age as fixed factor. AUC data was compared with general linear univariate analysis of variance with age as fixed factor. Sidak post hoc test was applied for pairwise comparisons if significant interaction was present. ***, *p* < 0.001, significant increase following supplement intake relative to baseline in both age groups. The older adults had decreased postprandial increase in pyridoxine (age effect *p* < 0.035, time effect *p* < 0,001) and PLP (age effect *p* = 0.009, time effect *p* < 0.001) but increased 4-pyridoxic acid (age × time, *p* = 0.002) than the younger adults (α, *p* < 0.05; β, *p* < 0.01; γ, *p* < 0.001, main age difference or difference with age × time interaction at particular time points).

**Figure 4:**
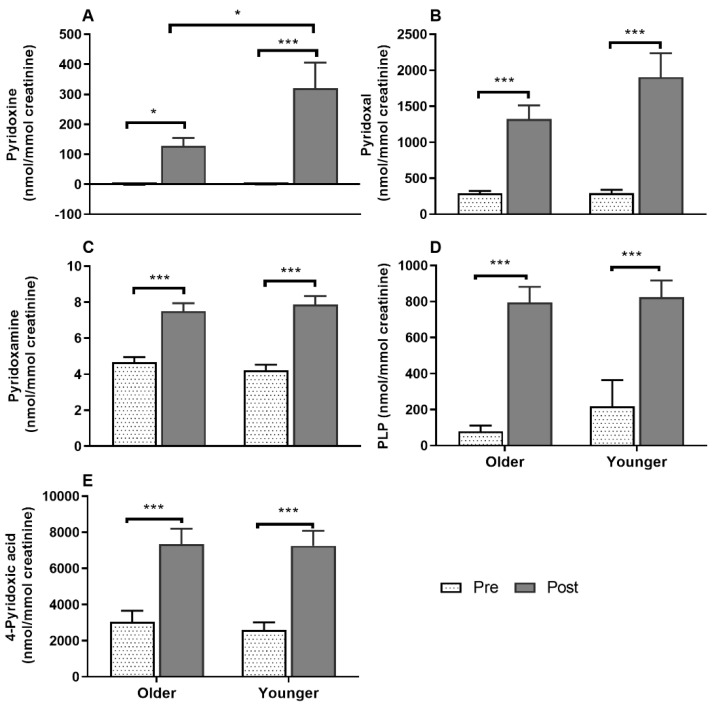
Urinary concentrations of the vitamers of vitamin B_6_, (**A**), pyridoxine (**B**), pyridoxal (**C**), pyridoxamine (**D**) pyridoxal 5′-phosphate (PLP), and (**E**) 4-pyridoxic acid at baseline (pre) and after (post) supplement ingestion in older and younger adults. Bar plots and error bars represent means and ± SEM, respectively, *n* = 20 in each age groups and concentrations are normalized for urinary creatinine (nmol/mmol creatinine). Data for pre and post supplement ingestion was compared using generalized linear repeated measures analysis of variance with time as within subject and age as between subject factor. Sidak post hoc test was applied for pairwise comparisons in case of an interaction. All the urinary vitamers increased following supplement ingestion compared to baseline (main time effect, *, *p* < 0.05; *** *p* < 0.001). A time x age interaction (*p* = 0.037) showed a greater urinary concentration of pyridoxine in younger compared to older adults post-supplement ingestion. There were no significant differences in urinary excretion of other vitamers between older and younger adults.

**Figure 5:**
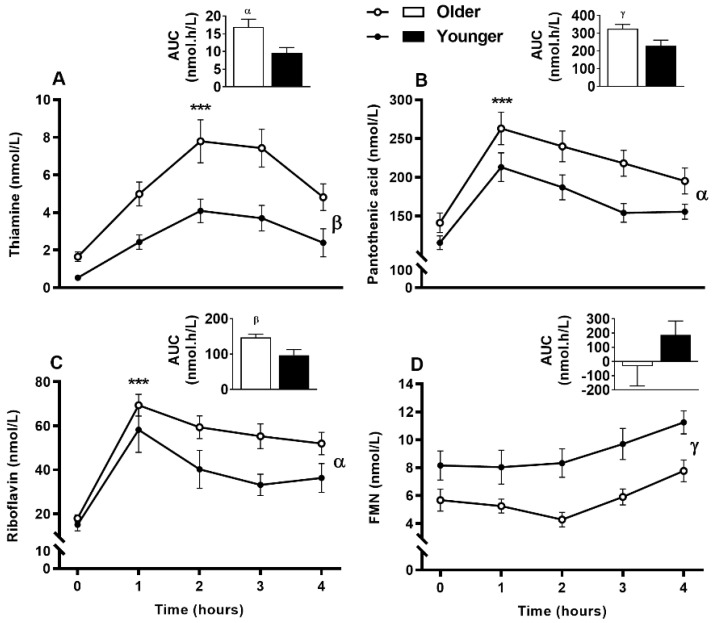
Plasma response of (**A**), thiamine (**B**), pantothenic acid (**C**), riboflavin, and its vitamer (**D**), flavin mononucleotide (FMN) at fasting (time, 0), hourly until four hours (time, 1, 2, 3, 4) and the four hour incremental area under the curve (AUC, represented by bar-graphs) following a single multivitamin and mineral supplement ingestion in older and younger adults. Data for multiple time points was compared using general linear mixed model with time as repeated and age as fixed factors. AUC data was compared with general linear univariate analysis of variance with age as fixed factor. ***, *p* < 0.001, significant increase following supplement intake relative to baseline in both age groups. The older adults had increased postprandial thiamine (age effect *p* = 0.001, time effect *p* < 0,001), pantothenic acid (age effect *p* = 0.008, time effect *p* < 0.001) and increased riboflavin (age effect *p* = 0.026, time effect *p* < 0.001) than the younger adults (α, *p* < 0.05; β, *p* < 0.01; γ, *p* < 0.001, main age difference).

**Figure 6:**
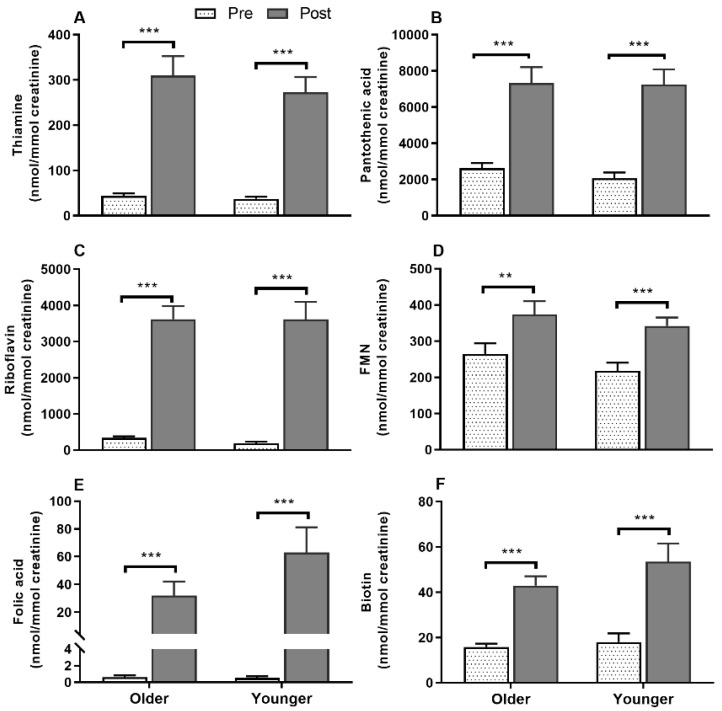
Urinary concentrations of (**A**) thiamine, (**B)**, pantothenic acid (**C**), riboflavin, the vitamer (**D**), flavin mononucleotide (FMN), (**E**) folic acid, and (**F**), biotin following supplement ingestion in older and younger adults. Bar plots and error bars represent means and ± SEM respectively, *n* = 20 in each age groups and concentrations were normalized for urinary creatinine (nmol/mmol creatinine). Data for pre and post supplement ingestion was compared using generalized linear repeated measures analysis of variance with time as within subject and age as between subject factor. Sidak post hoc test was applied for pairwise comparisons in case of an interaction. There were no interactions between any independent factors (age and time). There were main time effects (**, *p* < 0.01; ***, *p* < 0.001) for all the vitamins.

**Figure 7:**
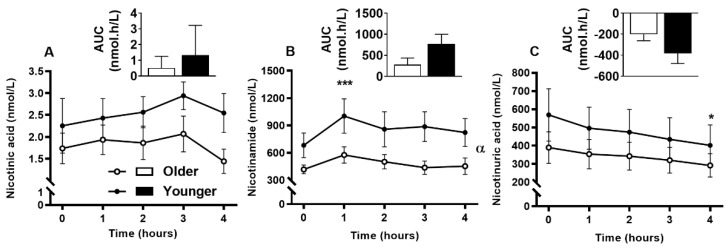
Plasma response of B_3_ vitamers, (**A**), nicotinic acid (**B**), nicotinamide, and (**C**), nicotinuric acid at baseline (fasting, time, 0), hourly until four hours (time, 1, 2, 3, 4) and the four hour incremental area under the curve (AUC, represented by bar-graphs) following a single multivitamin and mineral supplement ingestion in older and younger adults. Data for multiple time points was compared using general linear mixed model with time as repeated and age as fixed factors. AUC data was compared with general linear univariate analysis of variance with age as fixed factor. *, *p* < 0.05; ***, *p* < 0.001, significant change following supplement intake relative to baseline in both age groups. No significant difference in postprandial change between the age groups. Nicotinic acid concentration did not increase compared to baseline (time effect, *p* = 0.370), whereas nicotinamide increased (time effect, *p* < 0.001) with lower concentration in older than the younger adults (age effect α, *p* = 0.038) and nicotinuric acid decreased continuously throughout the 4-h postprandial period (time effect, *p* = 0.002).

**Figure 8:**
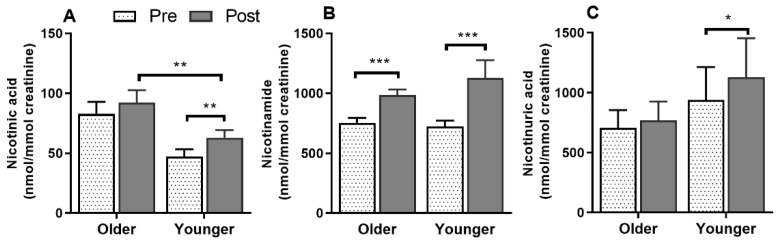
Urinary concentrations of the vitamers of vitamin B_3_, (**A**), nicotinic acid, (**B**), nicotinamide, and (**C**) nicotinuric acid at baseline (pre) and after (post) supplement ingestion in older and younger adults. Bar plots and error bars represent means and ± SEM respectively, *n* = 20 in each age groups and concentrations are normalized for urinary creatinine (nmol/mmol creatinine). Data for pre and post supplement ingestion was compared using generalized linear repeated measures analysis of variance with time as within subject and age as between subject factor. Sidak post hoc test was applied for pairwise comparisons in case of an interaction. There were no interactions between any independent factors (age and time). There were main time effects (*, *p* < 0.05; **, *p* < 0.01; ***, *p* < 0.001) for all the vitamers and a main age effect for nicotinic acid (**, *p* = 0.007).

Serum vitamin B_12_ (Figure 2A) was responsive to single supplement ingestion, as were almost all B-vitamins and vitamers, demonstrating increased postprandial concentrations. These included serum folate (Figure 2B); plasma and urinary B_6_-vitamers pyridoxine, pyridoxal, pyridoxamine, PLP and 4-PA (Figure 3 and Figure 4); plasma thiamine, riboflavin, FMN and pantothenic acid (Figure 5), urinary biotin and folic acid (Figure 6), plasma and urinary nicotinamide nicotinuric acid and urinary nicotinic acid (Figure 7 and Figure 8). Only postprandial plasma nicotinic acid concentration was unresponsive to supplement ingestion (Figure 7A).

### 3.6. Impact of Age on Acute Circulating Vitamin B_12_ and Folate Response to MVM Supplement Ingestion

Serum vitamin B_12_ concentrations were increased at 1 h following MVM ingestion (main time effect, *p* < 0.001). The older adults tended to have lower serum vitamin B_12_ (main age effect, *p* = 0.053) with a C_max_ of 493.88 ± 176.07 ng/mL compared to 601.26 ± 164.43 ng/mL in the younger adults. However, the postprandial serum vitamin B_12_ concentrations and overall appearance (AUC) were not different between older and younger subjects (Figure 2A). Sex specific difference between age groups were present (described below with sex-specific results).

### 3.7. Impact of Age on Acute Plasma and Urinary B_6_-Vitamer Responses to MVM Supplement Ingestion

All the B_6_-vitamers, pyridoxine, pyridoxal, pyridoxamine, PLP and 4-PA concentrations measured in both plasma (Figure 3A–E) and urine (Figure 4A–E) samples were responsive to MVM supplement ingestion. The older adults had a smaller postprandial AUC for both plasma pyridoxine and PLP concentrations than the younger group (*p* < 0.05; bar graphs, Figure 3A,D), and a larger AUC for the circulating excretory metabolite 4-PA (bar graph, Figure 3E). While postprandial plasma pyridoxine concentrations were not significantly different between older and younger individuals at any specific time point (Figure 3A, time x age interaction *p* > 0.05), younger subjects had higher postprandial pyridoxine concentration (age effect *p* = 0.035). Unlike younger adults, postprandial plasma PLP concentrations did not increase in the older group (Figure 3D, time x age interaction; *p* = 0.026; *p* > 0.05, older group baseline to postprandial time points), and remained lower than the younger group throughout the postprandial period (*p* < 0.05). In contrast plasma 4-PA concentration was higher in older than younger subjects from 3 h (Figure 3E, age x time, *p* = 0.002; *p* < 0.01). Only postprandial urinary concentrations of pyridoxine (time x age interaction *p* = 0.037; Figure 4) was higher in younger compared to older adults post-supplement ingestion. Although urinary concentrations of the other B_6_-vitamers (Figure 4) were not different between overall age groups, there were sex specific differences between age groups in pyridoxine and pyridoxal concentrations (detailed below in sex specific section).

### 3.8. Impact of Age on Acute Postprandial Plasma and Urinary Thiamine, Pantothenic Acid, Riboflavin, FMN, and Urinary Biotin Responses to Single MVM Supplement Ingestion

Postprandial plasma thiamine, pantothenic acid, and riboflavin concentrations were highly responsive to supplementation, while the riboflavin vitamer FMN showed a slower response (Figure 5). Interestingly, older adults had greater postprandial thiamine, pantothenic acid and riboflavin plasma concentrations than younger adults indicated by AUC (main age effect, *p* < 0.05), whereas FMN AUC did not differ between age groups (bar graphs, Figure 5A–D). Although thiamine (Figure 5A), pantothenic acid (Figure 5B), and riboflavin (Figure 5C) plasma concentrations were not significantly different between age groups at any specific time points (age x time interaction, *p* > 0.05), these vitamins were higher in the older group (age effect, *p* < 0.05). In contrast to riboflavin, plasma FMN (Figure 5D) showed a slow baseline to postprandial increase at 4 h (time effect, *p* < 0.001) and remained lower in older than younger adults (age effect, *p* = 0.001). However, postprandial urinary concentrations of these vitamins (Figure 6A–D) including folic acid (Figure 6E) and biotin (Figure 6F) were not different between any age groups.

### 3.9. Impact of Age on Acute Plasma and Urinary B_3_-Vitamer Responses to Single MVM Supplement Ingestion

There was no significant difference between the older and younger subjects in plasma AUC for any of the B_3_-vitamers (nicotinic acid, nicotinamide, and nicotinuric acid, bar graphs, Figure 7A–C). Although none of the B_3_-vitamers differed between age groups at any specific time points, older adults had lower plasma nicotinamide concentrations (age effect, *p* = 0.038, 7B), but no difference in nicotinic acid (age effect, *p* = 0.052, 7A) or nicotinuric acid concentrations (age effect, *p* = 0.326, 7C). The postprandial urinary concentration of B_3_-vitamers were not different between overall age groups (Figure 8A–C); however, nicotinuric acid differed between older and younger females (detailed below in sex specific section).

### 3.10. Sex-Specific Effects on Acute Circulating and Urinary B-Vitamin and Vitamer Responses to Supplement Ingestion

Several postprandial circulating B-vitamin responses demonstrated sex-specific differences within or between age groups, including vitamin B_12_, pyridoxal, riboflavin, thiamine, and nicotinuric acid (Figure S1). Postprandial serum vitamin B_12_ concentrations differed between age groups among the males and between sexes among the older group, with lower increases in older males (age × sex interaction; *p* = 0.007) than younger males (*p* = 0.002) and older females (*p* = 0.019).

Similarly, postprandial plasma B_6_-vitamers pyridoxal (Figure S1) and pyridoxamine, although not different between overall age groups, showed sex-specific age effects. Older females had lower postprandial plasma pyridoxal concentrations than younger females (*p* = 0.032; age × sex interaction, *p* = 0.043) although AUC was not different. Whereas no sex-specific differences were found in plasma pyridoxine, PLP and 4-PA concentrations were found across the age groups. Postprandial urinary excretion of pyridoxal and pyridoxine in younger females was also higher compared to older females and younger males (Figure S2; age x time x sex interactions *p* = 0.013 and *p* = 0.005, respectively; age comparison *p* = 0.001 and *p* = 0.002 and sex comparisons *p* < 0.001 each, respectively). Females, regardless of age, excreted more pyridoxamine, PLP and 4-PA compared to males (time x sex interaction *p* < 0.05 each, respectively; sex comparison *p* < 0.01 each, respectively).

Sex-specific differences were also seen for postprandial plasma (Figure S1) and urinary (Figure S2) riboflavin and thiamine responses and urinary pantothenic acid, folic acid and biotin (the latter two were measurable in urine but poorly detected in plasma). Younger males had lower plasma riboflavin responses (time × age × sex interaction, *p* = 0.033) than younger females while the plasma 4-h riboflavin AUC was, regardless of age, lower in males than females (sex effect, *p* = 0.033). Similarly, younger males had a lower plasma thiamine response (time × age × sex interaction *p* = 0.009) than older males (*p* = 0.012) at 1 and 2 h following the supplement; at 3 h, a similar age difference was found in females (*p* = 0.006). Interestingly, the younger males showed no significant increase in postprandial plasma thiamine concentrations at any timepoints relative to baseline (*p* > 0.05), whereas in all the other age and sex groups significant increases occurred at all 4 timepoints (*p* < 0.05). However, there was no impact of sex on the overall plasma thiamine AUC. Postprandial urinary concentrations of riboflavin, thiamine, pantothenic acid (Figure S2), folic acid, and biotin were greater in females (time x sex interaction *p* < 0.05) compared to males (*p* < 0.05). In urine, the riboflavin-vitamer FMN concentration was greater in females compared to males (sex effect *p* = 0.007).

Among the plasma B_3_-vitamers, a sex-specific effect was present only for postprandial nicotinuric acid, with lower concentrations in older females (age × sex interaction *p* = 0.015) than younger females (*p* = 0.017). However, the 4-h nicotinuric acid AUC was still lower in younger females (age × sex interaction, *p* = 0.041) compared to both younger males (*p* = 0.025) and older females (*p* = 0.011). The postprandial urinary excretion of both nicotinamide and nicotinuric acid were sex-dependent. Younger females had greater urinary nicotinamide excretion compared to younger males (*p* = 0.009) but not significantly different to older females (*p* = 0.060), whereas nicotinuric acid was greater in younger females compared to both younger males (*p* = 0.003) and older females (*p* = 0.005).

## 4. Discussion

Despite the greater risk for B-vitamin deficiency in older populations, the impact of aging on the postprandial response and dynamic regulation of individual vitamers is not well understood. The current study therefore investigated the postprandial response of a range of B-vitamins and their vitamers in older and younger subjects following a single MVM supplement ingestion in order to better understand the changes in absorption and metabolism of B-vitamins associated with aging. This study demonstrated that older men had a transiently different vitamin B_12_ response, with suppressed supplement-mediated increases compared to older females and younger males and females, but this did not impact the AUC over the 4 h studied. Older adults had lower postprandial plasma concentrations of the B_6_-vitamers pyridoxine and PLP, whereas they had higher 4-pyridoxic acid, thiamine, pantothenic acid, and riboflavin concentrations. While lower urinary pyridoxine excretion in the older adults matches circulating response, none of the other urinary metabolites corresponded with the plasma response. The decreased availability of ingested and active forms of B_6_-vitamers in older subjects suggests possible alterations in the absorption or subsequent metabolic interconversion of vitamin B_6_ in older people.

Serum vitamin B_12_ concentrations were lower in older males compared to both younger and older females both at fasting and postprandially. This is consistent with our hypothesis that acute postprandial vitamin B_12_ bioavailability is altered with increasing age. Age and sex-dependent variations in fasting vitamin B_12_ status have been previously reported, with lower vitamin B_12_ status reported in older adults [31,37], and in males [38], which is consistent with the current findings. Lower B_12_ has been attributed to genetic variations, as no significant associations with dietary habits or hormones could be established [38]. However, capturing the genetic variation was beyond the scope of the present study and not assessed in our population. Malabsorption of vitamin B_12,_ due to impaired gastrointestinal acid secretion is frequently reported in older populations [20,39] even when nutritional status is adequate [37,40]. However, although our fasting results suggest greater risk of B_12_ deficiency in older males, the total appearance (AUC) of postprandial vitamin B_12_ was not different between age groups. Hence, despite differences in fasting B_12_ status in older men, the current findings do not indicate that total acute appearance of B_12_ following a single MVM in these subjects is a contributing factor to low B_12_ status.

Of the B-vitamins and vitamers measured, the B_6_ vitamers responded most differently in older adults, suggesting alterations in postprandial B_6_ metabolism. Older adults showed a blunted increase in postprandial plasma pyridoxine and PLP; PLP is integral to the regulation of one-carbon metabolism and other diverse enzymatic reactions [2]. In contrast, the concentration of the metabolized end-product, 4-PA, which does not contribute to the co-enzyme functions of B_6_, was higher in plasma, but not urine in these subjects. The lower pyridoxine response in older adults could implicate either altered intestinal or hepatic metabolic conversion to the active coenzyme form PLP [41]; however, the absence of an age difference in postprandial plasma pyridoxal concentrations does not support the idea of inadequate enzymatic pyridoxine conversion. As urinary excretion of pyridoxine was lower in older adults, malabsorption, rather than greater excretion of absorbed pyridoxine, may be one possible explanation. Previous studies have found that factors such as inflammation and high protein intake alter circulating vitamin B_6_ distribution [42,43], such that PLP concentrations decrease during both acute and chronic adjuvant-arthritic inflammation [42] while increasing during high protein intake [43]. However, the enzymatic conversion of pyridoxine has not been previously reported with respect to the aging process outside of age-related altered protein metabolism [44]. Although low PLP has been implicated in systemic inflammatory responses including cardiovascular [45] and chronic kidney diseases [46], available evidence describes long term rather than postprandial implications. While in practice, these findings may suggest that older people have higher B_6_ substrate requirements, this would require further confirmation to understand the relationship between postprandial differences in availability and long-term requirements. Indeed, while acute responsiveness of the B vitamins studied here could be interpreted positively in the context of absorption or metabolic flexibility, it is similarly important to note that without an understanding of appropriate postprandial targets for these compounds, it is difficult to interpret whether age-related differences observed have any relevant physiological consequences.

Age-related enzymatic conversion and excretion differences may explain both a blunted pyridoxine and PLP response, and greater circulating 4-PA concentrations in the older subjects. Aldehyde oxidase 1 (AOX 1) facilitates this conversion [47]; as older adults had higher 4-PA in plasma post-ingestion, but not at baseline or in urinary excretion, it is possible that activity of AOX1 differed between older and younger subjects. While there are limited studies reporting age differences in B_6_ enzyme activity, including AOX1 [48,49,50], these findings align with several reports of decreased plasma PLP and increased 4-PA concentrations with advancing age [48,50], yet contradict another postprandial supplement study in men which found no age difference indices of vitamin B_6_ metabolism [19]. Equally conflicting, evidence from animal models has suggested vitamin B_6_ metabolizing enzymes are unaltered by age, despite decreased PLP concentrations [49], but others have demonstrated an age-related increase in pyridoxal kinase (PDXK) activity in humans [48,50], which would decrease pyridoxine but increase, rather than decrease, PLP concentrations. These authors concluded that the decline in PLP concentrations may be due to the changes in intermediary PLP-binding skeletal muscle protein metabolism during the metabolic switch from anabolism to catabolism during aging [48,51]. However, without further understanding alterations in intermediary enzymes, it is unclear whether the current observed differences in B_6_ vitamers are due to altered absorption or conversion. Regardless, given the importance of PLP as a coenzyme in diverse cellular mechanisms, including one carbon metabolism [5], the low postprandial response in circulating plasma in older adults may have functional impacts on successful aging.

Unlike the response of B_6_, the older age group demonstrated higher postprandial concentrations of some B-vitamins including thiamine, riboflavin, and pantothenic acid. The differences in thiamine and riboflavin responses between age groups were sex-specific to males, with younger males having lower concentrations than older males, while pantothenic acid differences were seen in both sexes. These contrasting findings contradict a simple alteration in absorption [52] or even cellular uptake [53]. A similar lack of age-related difference in circulating pantothenic acid concentrations was reported in a longer term intervention in an animal model [54], yet evidence for age-related differences in these vitamins is limited. The comparatively lower plasma response of both riboflavin and thiamine in younger males than the rest of the subjects was unexpected, and a non-significant but higher FMN in younger adults, regardless of gender, may suggest age-related differences in riboflavin conversion. However, this remains speculative as this study did not analyze FMN beyond 4 h post-ingestion. Nevertheless, a similar age-related difference in postprandial FMN response to a single riboflavin rich meal has been previously reported by our laboratory [35], suggesting altered riboflavin conversion to FMN. Other factors not assessed in the current study, such as physical activity, are known to increase requirements of thiamine, riboflavin and vitamin B_6,_ that may accelerate the cellular uptake of these vitamins, impacting circulating concentrations [53].

Urinary excretion of all the B_6_-vitamers and riboflavin, thiamine, and pantothenic acid was greater in females than males, with nicotinamide and nicotinuric acid concentrations also being greater in females, but only in the younger group, which contrasted with the absence of sex-specific differences in circulating concentrations of these vitamins. Others have similarly reported greater urinary excretion of thiamine, pantothenic acid, folate, and vitamin B_12_, but not of riboflavin and B_3_-vitamers in females following a 7-day controlled diet [55]. Healthy females may have lower requirements due to lower body weight and energy requirements than males [56], which may contribute to greater urinary excretion. Moreover, postprandial urinary creatinine concentration was lower in females, which would have amplified this difference as urinary B-vitamin measurements were corrected for urine output. However, more studies are required to understand the basis of these sex-specific differences in excretion, including the use of 24-h urinary collection to extend the data from the time-limited analysis in the current study. In particular, the lack of correlation between plasma and urinary concentrations highlights that measures of status and acute circulating response may differ from measures of elimination acutely, and may be useful in offering insight into differences in saturation of B vitamin uptake. However, like absorption, excretion of several vitamers (including thiamine and nicotinamide metabolites) has also been shown to be influenced by habitual intake [57], which was not controlled for in the current study.

The fasting concentrations of circulating thiamine, pantothenic acid, folate, and nicotinuric acid were different between older and younger subjects, which may have contributed to the age-related postprandial differences. Except for fasting thiamine and serum folate concentrations, which were higher in older adults, this relationship was sex-specific. Older compared to younger females had lower nicotinuric acid concentrations whereas males had lower fasting pantothenic acid than females of the older group. Although there have been reports of inadequate intake of B vitamins in older New Zealanders in community dwelling [58,59] and indigenous New Zealand Māori populations [60], and indications of inadequate status in residential care settings [61], these trends do not appear to be mirrored in this study population. Contrary to concerns regarding greater risk of thiamine deficiency with aging [62], and available assessments of circulating status [63], the present study found higher thiamine status in older subjects, corresponding with the trend in greater habitual thiamine intake. However, other age and sex specific differences, including fasting thiamine, riboflavin, and niacin concentrations were not in line with the habitual dietary assessments collected in this study. This could be related to complex interactions across dietary habits [64], food choices [65], or metabolism [66], as well as lifestyle factors such as physical activity [53], smoking, and alcohol consumption [67], which were not thoroughly explored in the current study. Longer term dietary assessment or other biomarker measures for specific B-vitamins status could elucidate this further, but were not a primary focus of this study.

The present study represented a simple but necessary approach to understand the key fundamental changes in the acute postprandial response to supplementation in aging. Although this study was not without limitations, these responses may be integral to the prevalence of low B-vitamin status found in older populations [2]. This was accomplished by a comprehensive analysis of B-vitamins including many vitamers, although further work should include plasma biotin and folate vitamers (5-methyltetrahydrofolate, tetrahydrofolate, and 5–10, methylenetetrahydrofolate), and should also consider the use of certified reference materials rather than plasma matrix QCs. Some commonly reported status measures of urinary excretion of vitamin B_12_ and folate were not reported (e.g., methylmalonic acid (MMA) and holotranscobalamin) [68], yet these are less appropriate for acute bioavailability as MMA is a functional biomarker and holotranscobalamin represents long term intake [69]. Changes in many tissue-specific vitamers, such as phosphorylated thiamine vitamers [70], may occur during a later phase of postprandial metabolism [71]; although these may not have been captured, additional work is required to determine their contribution to circulating pools. A further limitation of evaluating acute vitamin responses is the impact of habitual intestinal exposure in regulation of vitamin absorption; indeed, carrier-mediated mechanisms of B vitamins (including thiamine, riboflavin, and folate, but not vitamin B_6_) have been shown to be upregulated during periods of deficiency, and downregulated with excess supplementation [72,73]. It is unclear whether recent intake [74], which was uncontrolled prior to the study, or differences in habitual vitamin intake observed in the current study may have impacted absorption within the intestine differently across groups. There is also uncertainty regarding the actual vitamin content ingested, as this was based on the manufacturer’s information for the MVM tablet and food table estimates; both are prone to error, and may not reflect the ingested amounts [75]. Although inclusion of healthy participants may limit the generalizability of the results to typical aging populations with comorbidities including reduced gastrointestinal function [76], alcoholism [67], and other chronic diseases [77,78], this approach eliminated potential confounding factors that would have impacted the results. Impact of aging being the primary comparison, the study may not be sufficiently powered to confirm the sex-specific findings with low sample size, however, it provides preliminary data on the effect of sex on acute B-vitamin responses.

## 5. Conclusions

The present study demonstrated that although vitamin B_12_ response was not different between age groups, older males had lower B_12_ appearance in circulation from acute ingestion of a MVM supplement compared to younger males and older females. The significance for long-term B_12_ status in older males is unclear. There was a suppressed postprandial rise in the abundance of the active B_6_-vitamers, with higher excretory B_6_-vitamers. These data suggest a previously undescribed change in vitamin B_6_ absorption, metabolism, or interconversion in healthy older adults. Further studies investigating other related metabolites and enzymes involved in the B-vitamin metabolic pathway are required to understand the possible mechanisms. As the study may face limitations due to the small sample size, acute assessment period, lack of tissue measures in comparison to circulating concentrations, and uncertainty of the implications for adequate intake and status, the results should therefore be interpreted with caution. However, these current findings are suggestive of differences in the postprandial handling of differing members of the B-vitamin family and therefore contribute to the complexity of current understanding of the possible differing B-vitamin requirements for aging individuals.

## Figures and Tables

**Table 1:** Breakfast items consumed by the study participants.

Breakfast Items	Older Adults		Younger Adults	
	M (*n* = 10)	F (*n* = 10)	M (*n* = 10)	F (*n* = 10)
Toast (g)	76 ± 1.3	73.6 ± 2.4	75.9 ± 1.5	72.5 ± 0.7
Butter (g)	9.3 ± 0.4	9.5 ± 0.3	9.1 ± 0.6	8.4 ± 1.0
Honey (g) *	18.9 ± 0.6	19.2 ± 0.5	13.8 ± 2.1	17.3 ± 1.5
Applesauce (g)*	100 ± 0	100 ± 0	77.3 ± 12.1	82 ± 12.1
Orange Juice (mL)	242.1 ± 7.9	250 ± 0	250 ± 0	235 ± 15

Values are means ± standard error of the mean (SEM)s. All the food items were weighed before and after (unconsumed portions) eaten by the participants. *, significant difference in consumed portions between older and younger adults (*p* < 0.05 for the items indicated). M, males; F, females.

**Table 3:** Baseline characteristics and biochemistry of the study participants at fasting.

Variable	Older Adults (*n* = 20)		Younger Adults (*n* = 20)		Effect		
	M (*n* = 10)	F (*n* = 10)	M (*n* = 10)	F (*n* = 10)	Age	Sex	Age × Sex
Age (years)	71.1 ± 0.9	69.1 ± 0.7	23.3 ± 1.1	25.0 ± 0.5	<0.001 *	0.857	0.031 *
Weight (kg)	80.4 ± 4.1	69.4 ± 3.3	77.9 ± 3.6	62.7 ± 2.4	0.185	<0.001 *	0.539
Height (cm)	176.0 ± 2.3	165.4 ± 1.6	174.4 ± 1.6	165.3 ± 2.0	0.653	<0.001 *	0.696
BMI (kg/m^2^)	25.9 ± 1.0	25.3 ± 1.0	25.6 ± 1.2	23.0 ± 0.9	0.226	0.135	0.321
Waist circumference (cm)	90.5 ± 3.5	86.3 ± 3.1	83.1 ± 3.0	75.3 ± 2.0	0.004 *	0.051	0.545
Systolic BP (mmHg)	148 ± 4	128 ± 4	121 ± 3	110 ± 2	<0.001 *	<0.001 *	0.189
Diastolic BP (mmHg)	79 ± 3	73 ± 3	67 ± 1	71 ± 2	0.004 *	0.723	0.059
HOMA-IR	1.31 ± 0.32	1.47 ± 0.22	1.97 ± 0.41	1.38 ± 0.28	0.358	0.490	0.234
Fasting Serum Measures							
Glucose (mmol/L)	5.27 ± 0.18	4.74 ± 0.09	4.78 ± 0.15	4.49 ± 0.13	0.014 *	0.007 *	0.405
Insulin (µU/mL)	8.24 ± 1.65	6.51 ± 1.38	6.24 ± 1.46	7.39 ± 1.05	0.690	0.838	0.310
Cholesterol (mmol/L)	5.75 ± 0.39	6.61 ± 0.42	4.70 ± 0.33	4.57 ±0.27	<0.001 *	0.318	0.172
HDL-C (mmol/L)	1.40 ± 0.18	1.85 ± 0.09	1.47 ± 0.15	1.77 ± 0.14	0.960	0.006 *	0.559
LDL-C (mmol/L)	3.85 ± 0.39	4.40 ± 0.39	2.84 ± 0.28	2.63 ± 0.26	<0.001 *	0.624	0.263
Triglyceride (mmol/L)	1.51± 0.19	1.31 ± 0.21	1.06 ± 0.15	0.79 ± 0.07	0.005 *	0.156	0.803

Values are means ± SEMs. Data was compared using general linear multivariate analysis of variance with age and sex as fixed factors. * Significant main effects or interactions, *p* < 0.05. BMI, body mass index; BP, blood pressure; HOMA-IR, homeostatic model assessment of insulin resistance; HDL-C, high density lipoprotein cholesterol; LDL-C, low density lipoprotein cholesterol; M, males; F, females.

**Table 4:** Estimated habitual B-vitamin intake of the study participants from 3-day dietary intake records.

B-Vitamins	RDI^o^	Older Adults		RDI^y^	Younger Adults		Effect		
	M, F	M (*n* = 10)	F (*n* = 10)	M, F	M (*n* = 9) ^1^	F (*n* = 9) ^1^	Age	Sex	Age × Sex
Vitamin B_12_ (μg)	2.4	2.95 ± 0.4	3.12 ± 0.2	2.4	6.34 ± 1.1	4.31 ± 1.0	0.003 *	0.209	0.140
Total Folate (μg)		290.57 ± 62.5	377.45 ± 51.0		443.96 ± 105.8	168.40 ± 36.8	0.685	0.174	0.012 *
Folate, total DFE (μg)	400	437.81 ± 91.8	448.93 ± 83.3	400	465.11 ± 116.2	178.04 ± 37.8	0.172	0.123	0.097
Vitamin B_6_ (mg)	1.7, 1.5	2.33 ± 0.3	2.69 ± 0.3	1.3	3.83 ± 0.6	1.38 ± 0.2	0.782	0.005 *	<0.001 *
Thiamin (mg)	1.2, 1.1	1.60 ± 0.2	1.37 ± 0.1	1.2, 1.1	1.74 ± 0.4	0.77 ± 0.1	0.3	0.009 *	0.098
Riboflavin (mg)	1.3, 1.1 ^2^	1.88 ± 0.2	1.99 ± 0.2	1.3, 1.1	2.48 ± 0.4	1.20 ± 0.2	0.74	0.036 *	0.013 *
Niacin (mg)		19.35 ± 2.4	18.30 ± 1.5		32.66 ± 4.3	12.48 ± 2.2	0.18	<0.001 *	0.001 *
Niacin E (mg)	16, 14	36.88 ± 3.1	33.59 ± 2.2	16, 14	63.59 ± 7.3	24.84 ± 3.4	0.044 *	<0.001 *	<0.001 *

Foodworks software was used to extract the nutrient composition from the New Zealand food composition database. Data was compared using general linear multivariate analysis of variance with age and sex as fixed factors. Sidak post hoc test was applied if significant age × sex interaction was present. * Significant main effects or interactions, *p* < 0.05 DFE, dietary folate equivalent; Niacin E, niacin equivalent; RDI, recommended dietary intake; o, older; y, younger; M, males; F, females. ^1^ Dietary intake records for one younger male and one female could not be obtained. ^2^ RDI presented for 51–70 years. RDI for >70 years is 1.6 and 1.3 mg for males and females, respectively.

## Supplementary Materials

The following are available online at https://www.mdpi.com/2072-6643/12/11/3529/s1, Table S1: Urine output and creatinine concentration of the study participants at fasting and 1–4 h following multivitamin and mineral supplement ingestion; Figure S1: Postprandial circulating B-vitamin concentrations with sex-specific effects following supplement ingestion; Figure S2: Sex-specific effects on the urinary concentrations of B-vitamins and vitamers following supplement ingestion.
