# Supplementary material for: Circulatory and Urinary B-Vitamin Responses to Multivitamin Supplement Ingestion Differ between Older and Younger Adults
Source: Nutrients. 2020 Nov 17;12(11):3529. doi: 10.3390/nu12113529 (PMC7698360; doi:10.3390/nu12113529)
Supplement: Supplementary file 1 [file nutrients-12-03529-s001.pdf]

Supplemental Tables

Table S1: Urine output and creatinine concentration of the study participants at fasting and 1-4 h following multivitamin and mineral supplement ingestion**.**

Urine	Old		Younger		*P* value		
	M	F	M	F	Age	Sex	Age x sex
Volume fasting (g)	41.1 ± 2.3	45.1 ± 4.3	46.9 ± 5.6	32.1 ± 5.7	0.456	0.267	0.058
Volume 1-4 h (g)	408.7 ± 88	489.9 ± 113.7	469 ± 147.2	508.9 ± 83.4	0.735	0.606	0.860
Creatinine fasting (mmol/L)	11.7 ± 0.9	8.4 ± 1.5	18.9 ± 3.6	17.5 ± 3.7	0.005*	0.401	0.734
Creatinine 1-4 h (mmol/L)	7.5 ± 1.2	5 ± 1.7	8.7 ± 2.2	3.2 ± 0.5	0.813	0.012*	0.328

M: male; F: female. *, significant main age or sex effects, $P < 0.05$.

Values are presented as mean ± SEM (n=20 in each age group with 10 males and 10 females each.)

Statistical analysis was performed using general linear univariate model with age and sex as fixed factors; α $P < 0.05$.

Table S2. Baseline B-vitamin and vitamer concentration in circulation and urine of the study participants measured at fasting.

	Older adults		Younger adults		Effect		
**Circulation**	M (n=10)	F (n=10)	M (n=10)	F (n=10)	Age	Sex	Age x sex
Vitamin B_{12}[1]	351.9 ± 31.9[a]	535.4 ± 64.7[b]	569.6 ± 29.7[b]	473.2 ± 53.4	0.109	0.363	0.005[†]
Folate[1]	16.5 ± 3.2	15.9 ± 1.4	10.1 ± 1.1	8.9 ± 1.1	0.001[*]	0.689	0.878
Pyridoxine	0.2 ± 0.1	0.2 ± 0.0	0.3 ± 0.2	0.1 ± 0.0	0.506	0.333	0.352
Pyridoxal	9.2 ± 3.2	4.6 ± 1.5	10.1 ± 1.5	11.6 ± 2.9	0.108	0.520	0.217
Pyridoxamine	1.0 ± 0. 2	0.9 ± 0.3	1.2 ± 0.2	0.6 ± 0.2	0.988	0.116	0.247
PLP	2.1 ± 1.6	1.0 ± 0.3	1.1 ± 0.5	2.7 ± 1.9	0.822	0.864	0.298
4-pyridoxic acid	20.7 ± 5.6	21.9 ± 2.2	16.6 ± 2.3	17.1 ± 3.2	0.222	0.819	0.916
Thiamine	1.3 ± 0.4	2 ± 0.3	0.5 ± 0.1	0.6 ± 0.2	0.001[*]	0.161	0.276
Pantothenic acid	113.3 ± 4.1[a]	169.5 ± 22.1[b]	124.3 ± 13.1	106.6 ± 12.3[a]	0.080	0.189	0.014[†]
Riboflavin	18.1 ± 2.3	17.9 ± 2.5	10.1 ± 1.1	20.1 ± 5.1	0.352	0.127	0.108
FMN	4.4 ± 0.9	6.9 ± 1.2	7.7 ± 1.6	8.6 ± 1.5	0.064	0.186	0.520
Nicotinic acid	1.7 ± 0.4	2.1 ± 0.4	2.5 ± 1.2	2.3 ± 0.4	0.512	0.896	0.667
Nicotinamide	331.1 ± 63.7	503.6 ± 68.4	641.3 ± 266.7	724.2 ± 74.5	0.078	0.388	0.761
Nicotinuric acid	582.4 ± 145.6	196.7 ± 48.2[a]	358.6 ± 90.1	778.4 ± 262.5[b]	0.267	0.915	0.016[†]
**Urine**							
Folic acid	0.4 ± 0.1	0.9 ± 0.4	0.3 ± 0.1	0.8 ± 0.4	0.715	0.093	0.984
Pyridoxine	0.1 ± 0.0	0.1 ± 0.0	0.1 ± 0.0	0.1 ± 0.0	0.116	0.395	0.501
Pyridoxal	250.8 ± 45.3	332.7 ± 39.5	244.8 ± 50.7	343.5 ± 71.0	0.964	0.097	0.875
Pyridoxamine	4.5 ± 0.3	4.9 ± 0.5	3.4 ± 0.3	5.1 ± 0.4	0.341	0.089	0.280
PLP	32.2 ± 15.4	125.9 ± 60.2	13.4 ± 6.7	423.2 ± 281.5	0.234	0.01[*]	0.091
4-pyridoxic acid	2316.4 ± 834.0	1938.8 ± 174.6	1451.7 ± 149.2	2195.6 ± 546.3	0.556	0.722	0.280
Thiamine	40.5 ± 7.9	46.8 ± 8.0	40.5 ± 6.1	34.0 ± 6.9	0.383	0.994	0.385
Pantothenic acid	1890.9 ± 85.5	3363.5 ± 454.4	1697.2 ± 391.0	2433.0 ± 500.7	0.161	0.008[*]	0.355
Riboflavin	323.1 ± 56.7	352.4 ± 63.2	149.9 ± 27.8	227.5 ± 74.8	0.015[*]	0.365	0.681
FMN	241.8 ± 32.1	288.1 ± 49.5	187.9 ± 16.9	248.0 ± 41.2	0.212	0.159	0.853
Nicotinic acid	65.4 ± 5.7	100.0 ± 18.2	34.6 ± 3.8	59.6 ± 10.4	0.003[*]	0.01[*]	0.664
Nicotinamide	772.5 ± 30.6	731.4 ± 81.7	611.6 ± 45.0[a]	834.9 ± 71.7[b]	0.64	0.143	0.036[†]
Nicotinuric acid	995.9 ± 254.6	414.4 ± 107.9[a]	336.5 ± 96.8[a]	1540.2 ± 478.8[b]	0.412	0.275	0.003[†]
Biotin	12.3 ± 0.6	19.5 ± 2.2	13.6 ± 1.9	22.4 ± 7.2	0.589	0.047[*]	0.851

Values are means ± SEMs of concentrations in circulation (nmol/L) or urine (nmol/mmol creatinine), except otherwise stated. Circulatory [1]vitamin B_{12} (pg/mL) and folate (mg/mL) were measured in serum, the rest in plasma samples folic acid and biotin were only detected in urine. Data were compared using general linear multivariate analysis of variance with age and sex as fixed factors. Sidak post-hoc test was applied for pairwise comparisons if significant age x sex interaction was present. Significant age x sex interaction ($p < 0.05$) is indicated by"†", main effects are indicated by "*". Different superscript letters within a row indicate significantly different values between groups ($p < 0.05$). M, males; F, females.

Supplemental Figure 1.

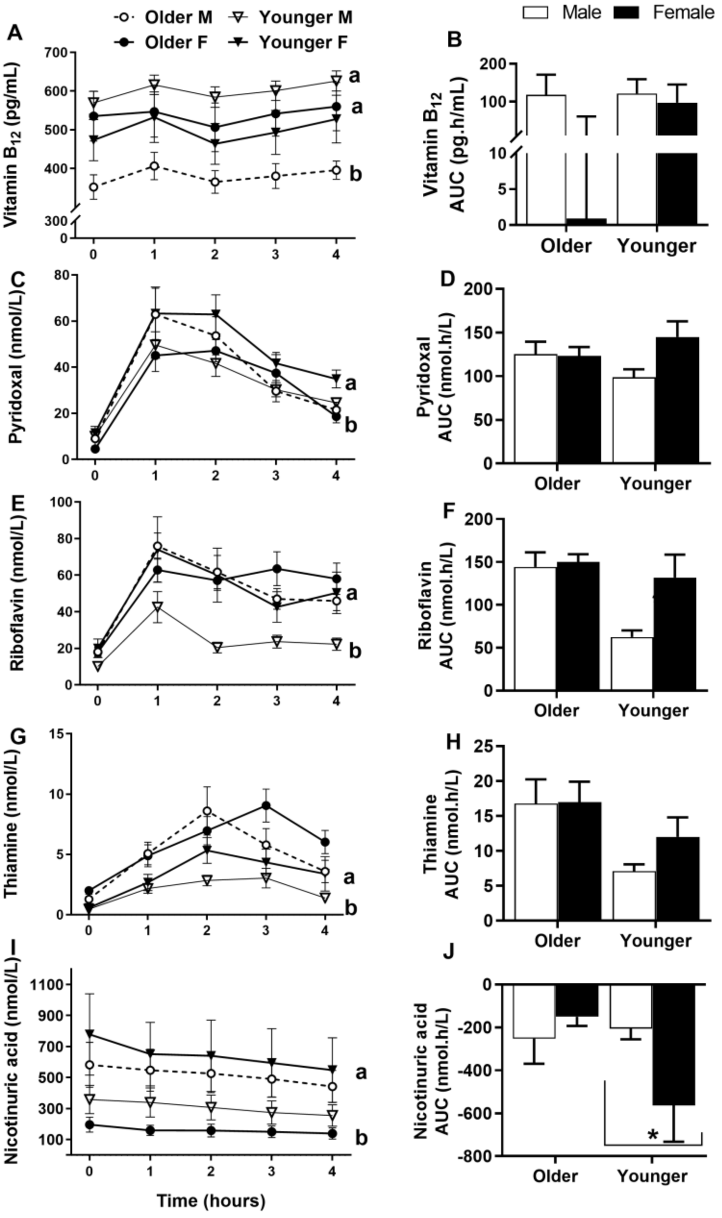

Figure S1. Postprandial circulating B-vitamin concentrations with sex-specific effects in the older and younger adults following a multivitamin and mineral supplement ingestion. The panel on the left represents the time response concentration curves at each study time points including at fasting (Time=0 hour) and the right panel are the incremental area under the curves (iAUC) for the respective vitamins, (AB) serum vitamin B_{12}, (CD) pyridoxal, (EF) riboflavin, (GH) thiamine and (IJ) nicotinuric acid. Different alphabetical letters indicate a statistically significant difference (P = 0.05) between the groups indicated and asterisks (*) indicate the difference in the 4-hour iAUC between age or sex groups.

Supplemental Figure 2.

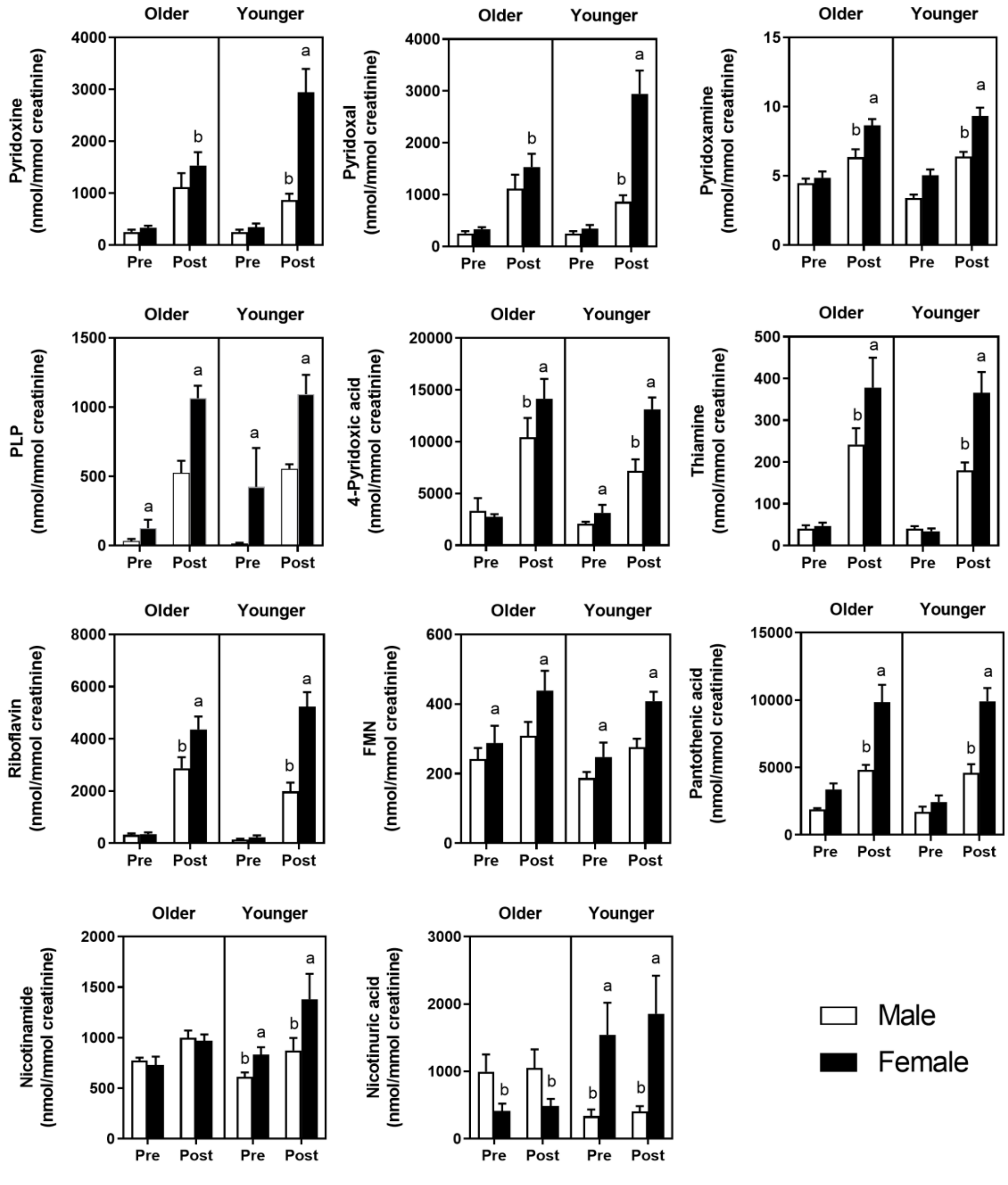

Figure S2. Sex - specific effects on the urinary concentrations of B-vitamins and vitamers before (pre) and after (post) multivitamin supplement ingestion. Different alphabetical letters indicate significant differences with interactions (time x age x gender, $P < 0.05$) between or within age groups as indicated, whereas similar letters indicate a main sex effect, P=0.05) on vitamin concentration.
